# A nonlinear correlation between the serum uric acid to creatinine ratio and the prevalence of hypertension: a large cross-sectional population-based study

**DOI:** 10.1080/0886022X.2023.2296002

**Published:** 2024-01-08

**Authors:** Ru Wang, Shuxing Wu, Jing Wang, Wenting Li, Jian Cui, Zhuhua Yao

**Affiliations:** a Department of Cardiology, Tianjin Union Medical Center, Tianjin, China; b The Institute of Translational Medicine, Tianjin Union Medical Center of Nankai University, Tianjin, China

**Keywords:** Serum uric acid to creatinine ratio, serum uric acid, creatinine, hypertension, nonlinearity

## Abstract

**Objective:**

To explore the relationship between the serum uric acid to creatinine (UA/Cr) ratio and the prevalence of hypertension.

**Methods:**

In this cross-sectional study, we included 8571 individuals from the China Health and Nutrition Survey. Logistic regression analysis and restricted cubic spline (RCS) were used to analyze the relationship between the UA/Cr ratio and hypertension.

**Results:**

Compared with individuals without hypertension, individuals with hypertension had higher UA/Cr ratios. Multivariate logistic regression analysis showed that a higher UA/Cr ratio was closely related to a higher risk of hypertension (as a continuous variable, OR: 1.054, 95% CI: 1.014-1.095, *p* = 0.007; as a categorical variable, Q3 vs. Q1, OR: 1.183, 95% CI: 1.011-1.384, *p* = 0.035; Q4 vs. Q1, OR: 1.347, 95% CI: 1.146-1.582, *p* < 0.001). Subgroup analysis revealed that the correlation between the UA/Cr ratio and hypertension risk was stable in all subgroups except for the subgroup with diabetes and the subgroup with a BMI ≥ 28 kg/m^2^ (*p* < 0.05). Sensitivity analysis confirmed the robustness of the relationship between a higher UA/Cr ratio and a higher risk of hypertension (*p* < 0.05). The RCS showed that the UA/Cr ratio was nonlinearly related to hypertension risk. Further threshold effect showed that only a UA/Cr ratio less than 5.0 was related to hypertension risk (OR: 1.178, 95% CI: 1.086-1.278, *p* < 0.001), and the 2-piecewise linear regression model was superior to the 1-line linear regression model (*p* < 0.05).

**Conclusion:**

The UA/Cr ratio was associated with the prevalence of hypertension.

## 1. Introduction

Serum uric acid (UA) is the final product of purine metabolism. UA is mainly excreted by the kidney, and its serum concentration can usually be affected by renal function [1]. Current studies have shown that the UA level is closely related to diabetes, coronary heart disease, stroke and mortality risk [2–5]. In addition to UA, serum creatinine (Cr) is not only the main index of renal function but also directly reflects the state of kidney disease and has been proven to be closely related to a high risk of hypertension, cardiovascular disease and death [6–9]. However, these two renal function indices are prone to fluctuations and have limited value in predicting disease. Therefore, considering the influence of renal function, UA and Cr are combined to evaluate disease incidence. Several studies have shown that the UA/Cr ratio is better than a single index for predicting chronic kidney disease (CKD) [10]. In addition, some studies have revealed correlations between the UA/Cr ratio and renal disease progression, metabolic syndrome, nonalcoholic fatty liver disease (NAFLD), atrial fibrillation recurrence, stroke recurrence and mortality [11–17], but the correlation of the UA/Cr ratio with hypertension risk has not been well studied.

Therefore, according to the current research background, we explored the relationship between the UA/Cr ratio and the prevalence of hypertension among people from the China Health and Nutrition Survey (CHNS) to determine the importance of the UA/Cr ratio in the risk assessment of hypertension.

## 2. Subjects, materials and methods

### 2.1. Study population

**Figure 1.**
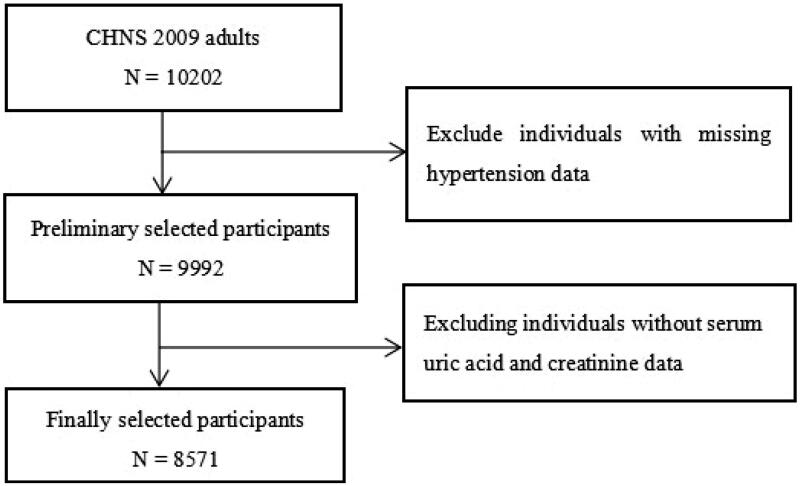
Flow chart of the study population. CHNS, China Health and Nutrition Survey.

In this large cross-sectional study, all participants were from the CHNS (2009). As shown in Figure 1, after excluding individuals without serum uric acid, creatinine and hypertension data, 8571 individuals were ultimately included in the analysis. The study protocol was approved by the institutional review committees at the University of North Carolina at Chapel Hill and the National Institute of Nutrition and Food Safety, Chinese Center for Disease Control and Prevention, and was in line with the Helsinki Declaration of 1975. All participants signed a written informed consent form.

### 2.2. Data collection and definitions

In this study, we selected several covariates for statistical analysis, including demographic variables, complication and drug treatment variables, and biomarker variables. Smoking status was divided into three groups: now, ever and never. Diabetes status was defined as a fasting plasma glucose (FPG) level ≥ 7.0 mmol/L, a hemoglobin A1c (HbA1c) level ≥ 6.5%, the use of hypoglycemic drugs, or a history of diabetes. Hypertension was defined as hypertension diagnosed by doctors, a current systolic blood pressure (SBP)/diastolic blood pressure (DBP) ≥ 140/90 mmHg or the use of antihypertensive drugs. CKD was defined as an estimated glomerular filtration rate (eGFR) < 60 mL/min/1.73 m^2^ according to published literature [18]. All blood marker levels were determined by trained professionals in standardized laboratories. In this study, the UA/Cr ratio was calculated as follows: UA/Cr = UA (µmol/L)/Cr (µmol/L).

### 2.3. Statistical analysis

In this study, categorical variables were expressed as frequencies (percentages), and the chi-square test was used to evaluate the differences in categorical variables between groups. Continuous variables with a normal distribution were expressed as the mean ± standard deviation, and the independent sample T test was used to evaluate the differences in continuous variables between groups. Continuous variables that did not conform to a normal distribution were represented by the median (the first quartile and the third quartile), and the Mann–Whitney U test was used to evaluate the differences in continuous variables between groups. The covariates with *p* < 0.1 in univariate analysis were subsequently selected for logistic regression analysis to construct three models: Model 1 did not adjust for covariates; Model 2 adjusted for only age and sex; and Model 3 adjusted for age, sex, smoking status, diabetes, hypoglycemic drug use, body mass index (BMI), triglycerides, total cholesterol, low-density lipoprotein cholesterol, high-density lipoprotein cholesterol, apolipoprotein B, FPG, HbA1c, high-sensitivity C-reactive protein and the eGFR. All individuals were subsequently divided into several subgroups to continue to explore the hierarchical association between the UA/Cr ratio and hypertension (age: < 60 years or ≥ 60 years; sex: male or female; diabetes: yes or no; CKD: yes or no; BMI: < 28 kg/m^2^ or ≥ 28 kg/m^2^). Individuals with an eGFR < 30 mL/min/1.73 m^2^ were excluded from the sensitivity analysis to verify the robustness of the correlation between the UA/Cr ratio and hypertensio. In addition, we also used a restricted cubic spline (RCS) to explore the potential nonlinearity and inflection point between the UA/Cr ratio and hypertension. If nonlinearity was tested, a 2-piecewise logistic regression model was constructed to evaluate the threshold effect of the UA/Cr ratio on hypertension, and a log-likelihood ratio test was performed to compare the 2-piecewise logistic regression model with the 1-line logistic regression model. All the statistical analyses were conducted in R 3.6.3, and *p* < 0.05 was used to indicate statistical significance.

## 3. Results

### 3.1. Baseline characteristics

Compared with the group without hypertension, the group with hypertension was older, had higher percentages of participants who were male, ever smokers, diabetes patients and hypoglycemic drug users, had higher BMI, SBP, DBP, triglyceride, total cholesterol, low density lipoprotein cholesterol, apolipoprotein B, Cr, UA, FPG, HbA1c, high-sensitivity C-reactive protein and UA/Cr values, and had lower high-density lipoprotein cholesterol and eGFR values (*p* < 0.05) (Table 1).

**Table 1.** Baseline characteristics of participants stratified by the hypertension.

	Total population	Non-hypertension	Hypertension	P value
Age, years	50.44 ± 15.03	46.96 ± 14.42	59.16 ± 12.82	< 0.001
Sex, male, n (%)	4033 (47.10%)	2817 (46.00%)	1216 (49.80%)	0.001
Smoking status, n (%)				< 0.001
Now	2378 (27.80%)	1702 (27.80%)	676 (27.70%)	
Ever	282 (3.30%)	155 (2.50%)	127 (5.20%)	
Never	5908 (69.00%)	4270 (69.70%)	1638 (67.10%)	
Diabetes, n (%)	927 (10.80%)	458 (7.50%)	469 (19.20%)	< 0.001
Hypoglycemic drugs, n (%)	217 (2.50%)	75 (1.20%)	142 (5.80%)	< 0.001
Body mass index, kg/m^2^	23.38 ± 3.48	22.86 ± 3.24	24.68 ± 3.71	< 0.001
Systolic blood pressure, mmHg	125.01 ± 19.06	116.10 ± 11.19	144.77 ± 18.01	< 0.001
Diastolic blood pressure, mmHg	80.30 ± 11.17	75.59 ± 7.51	90.72 ± 10.88	< 0.001
Triglyceride, mmol/L	1.26 (0.86, 1.96)	1.17 (0.80, 1.80)	1.51 (1.01, 2.33)	< 0.001
Total cholesterol, mmol/L	4.86 ± 1.01	4.77 ± 0.98	5.11 ± 1.02	< 0.001
LDL‑C, mmol/L	2.98 ± 0.98	2.91 ± 0.96	3.16 ± 1.02	< 0.001
HDL‑C, mmol/L	1.44 ± 0.49	1.45 ± 0.44	1.41 ± 0.60	0.004
Apolipoprotein A1, g/L	1.16 ± 0.38	1.15 ± 0.35	1.17 ± 0.45	0.055
Apolipoprotein B, g/L	0.91 ± 0.27	0.88 ± 0.26	0.99 ± 0.28	< 0.001
CR, µmol/L	87.60 ± 23.14	86.00 ± 21.33	91.62 ± 26.73	< 0.001
UA, µmol/L	308.77 ± 105.63	299.08 ± 104.00	333.07 ± 105.78	< 0.001
Fasting plasma glucose, mmol/L	5.41 ± 1.47	5.26 ± 1.29	5.78 ± 1.79	< 0.001
Hemoglobin A1c, %	5.63 ± 0.92	5.53 ± 0.79	5.89 ± 1.14	< 0.001
Hs-CRP, mg/L	1.00 (0, 2.00)	1.00 (0, 2.00)	2.00 (1.00, 3.00)	< 0.001
eGFR, ml/min/1.73 m^2^	81.89 ± 17.84	84.16 ± 17.92	76.21 ± 16.32	< 0.001
UA/Cr	3.56 ± 1.41	3.51 ± 1.50	3.70 ± 1.14	< 0.001

Data were expressed as mean ± SD, median (interquartile range), or n (%). LDL-C, low-density lipoprotein cholesterol; HDL-C, high-density lipoprotein cholesterol; CR, creatinine; UA, uric acid; Hs-CRP, high-sensitivity C-reactive protein; eGFR, estimated glomerular filtration rate.

### 3.2. Multivariate logistic regression analysis

Multivariate logistic regression analysis revealed that a higher UA/Cr ratio was still closely related to a higher risk of hypertension after adjustment for age, sex, smoking status, diabetes, hypoglycemic drug use, and BMI, triglyceride, total cholesterol, low-density lipoprotein cholesterol, high-density lipoprotein cholesterol, apolipoprotein B, FPG, HbA1c, high-sensitivity C-reactive protein and eGFR values (as a continuous variable, OR: 1.054, 95% CI: 1.014-1.095, *p* = 0.007; as a categorical variable, Q3 vs. Q1, OR: 1.183, 95% CI: 1.011-1.384, *p* = 0.035; Q4 vs. Q1, OR: 1.347, 95% CI: 1.146-1.582, *p* < 0.001) (Table 2).

**Table 2.** Multivariate logistic regression analysis of association between the UA/Cr ratio with hypertension.

	Model 1		Model 2		Model 3	
	OR (95% CI)	P value	OR (95% CI)	P value	OR (95% CI)	P value
Q1	Ref	–	Ref	–	Ref	–
Q2	1.151 (1.002, 1.323)	0.047	1.160 (0.998, 1.348)	0.052	1.085 (0.927, 1.268)	0.309
Q3	1.415 (1.235, 1.622)	< 0.001	1.485 (1.281, 1.722)	< 0.001	1.183 (1.011, 1.384)	0.035
Q4	1.736 (1.518, 1.984)	< 0.001	1.964 (1.697, 2.273)	< 0.001	1.347 (1.146, 1.582)	< 0.001
P for trend	–	< 0.001	–	< 0.001	–	0.002
UA/Cr^a^	1.123 (1.077, 1.171)	< 0.001	1.161 (1.109, 1.217)	< 0.001	1.054 (1.014, 1.095)	0.007

^a^ The OR was examined by per 1-unit increase of UA/Cr. Model 1: unadjusted; Model 2: adjusted for age and sex; Model 3: adjusted for variables included in Model 2 and smoking, diabetes, hypoglycemic drugs, body mass index, triglyceride, total cholesterol, low-density lipoprotein cholesterol, high-density lipoprotein cholesterol, apolipoprotein B, fasting plasma glucose, hemoglobin A1c, high-sensitivity C-reactive protein and eGFR. UA, uric acid; CR, creatinine; eGFR, estimated glomerular filtration rate; OR, odd ratio; CI, confidence interval.

### 3.3. Subgroup analysis and sensitivity analysis

Subgroup analysis of patients revealed that the correlation between the UA/Cr ratio and hypertension risk was stable in the < 60 years, ≥ 60 years, male, female, nondiabetic, CKD, non-CKD and BMI < 28 kg/m^2^ subgroups (*p* < 0.05) (Table 3). As shown in Table S1, after excluding individuals with an eGFR < 30 mL/min/1.73 m^2^, the baseline characteristics and differences between the two groups were largely similar to those shown in Table 1. Sensitivity analysis once again confirmed the robustness of the relationship between a higher UA/Cr ratio and a higher risk of hypertension; that is, the UA/Cr ratio remained closely associated with a higher risk of hypertension after we adjusted for confounding variables and excluded participants with an eGFR < 30 mL/min/1.73 m^2^ (as a continuous variable, OR: 1.053, 95% CI: 1.013-1.094, *p* = 0.008; as a categorical variable, Q3 vs. Q1, OR: 1.184, 95% CI: 1.012-1.386, *p* = 0.035; Q4 vs. Q1, OR: 1.347, 95% CI: 1.146-1.583, *p* < 0.001) (Table 4).

**Table 3.** Subgroups analyses for the association between the UA/Cr ratio and the hypertension.

	Q1	Q2	Q3	Q4		
	OR (95% CI)	OR (95% CI)	OR (95% CI)	OR (95% CI)	P for trend	P for interaction
Age						0.136
< 60 years	Ref	1.092 (0.888, 1.344)	1.253 (1.020, 1.538)*	1.257 (1.010, 1.563)*	0.105	
≥ 60 years	Ref	1.079 (0.849, 1.371)	1.060 (0.832, 1.352)	1.474 (1.136, 1.913)**	0.016	
Sex						0.506
Male	Ref	1.211 (0.957, 1.533)	1.229 (0.973, 1.553)	1.349 (1.056, 1.723)*	0.119	
Female	Ref	0.985 (0.796, 1.218)	1.111 (0.894, 1.381)	1.314 (1.042, 1.658)*	0.064	
Diabetes						0.798
Yes	Ref	1.211 (0.770, 1.904)	1.329 (0.864, 2.044)	1.322 (0.840, 2.081)	0.581	
No	Ref	1.050 (0.888, 1.242)	1.133 (0.956, 1.342)	1.295 (1.087, 1.544)**	0.022	
CKD						0.061
Yes	Ref	1.616 (1.041, 2.507)*	1.929 (1.200, 3.100)**	2.127 (1.218, 3.716)**	0.014	
No	Ref	1.046 (0.884, 1.238)	1.136 (0.961, 1.343)	1.288 (1.088, 1.524)**	0.015	
BMI						0.329
< 28 kg/m^2^	Ref	1.123 (0.952, 1.324)	1.166 (0.987, 1.378)	1.385 (1.165, 1.647)***	0.003	
≥ 28 kg/m^2^	Ref	0.920 (0.528, 1.601)	1.454 (0.864, 2.446)	1.245 (0.738, 2.101)	0.211	

The multivariate adjusted model used in the subgroups analysis consisted of all covariates used in model 3 in Table 2 except for the variable (as a categorical variable) that was used for stratification. The interaction of the UA/Cr ratio and variables used for stratification was examined by likelihood ratio tests.

*p < 0.05, **p < 0.01, ***p < 0.001. UA, uric acid; CR, creatinine; CKD, chronic kidney disease; BMI, body mass index; OR, odd ratio; CI, confidence interval.

**Table 4.** Association between the UA/Cr ratio with hypertension after excluding participants with eGFR < 30 mL/min/1.73 m^2^.

	Model 1		Model 2		Model 3	
	OR (95% CI)	P value	OR (95% CI)	P value	OR (95% CI)	P value
Q1	Ref	–	Ref	–	Ref	–
Q2	1.171 (1.018, 1.346)	0.027	1.170 (1.006, 1.360)	0.042	1.090 (0.931, 1.275)	0.284
Q3	1.437 (1.253, 1.648)	< 0.001	1.495 (1.289, 1.735)	< 0.001	1.184 (1.012, 1.386)	0.035
Q4	1.765 (1.543, 2.018)	< 0.001	1.981 (1.711, 2.294)	< 0.001	1.347 (1.146, 1.583)	< 0.001
P for trend	–	< 0.001	–	< 0.001	–	0.003
UA/Cr^a^	1.130 (1.083, 1.179)	< 0.001	1.165 (1.112, 1.221)	< 0.001	1.053 (1.013, 1.094)	0.008

^a^ The OR was examined by per 1-unit increase of UA/Cr. Model 1: unadjusted; Model 2: adjusted for age and sex; Model 3: adjusted for variables included in Model 2 and smoking, diabetes, hypoglycemic drugs, body mass index, triglyceride, total cholesterol, low-density lipoprotein cholesterol, high-density lipoprotein cholesterol, apolipoprotein B, fasting plasma glucose, hemoglobin A1c, high-sensitivity C-reactive protein and eGFR. UA, uric acid; CR, creatinine; eGFR, estimated glomerular filtration rate; OR, odd ratio; CI, confidence interval.

### 3.4. RCS and threshold effect analyses

**Figure 2.**
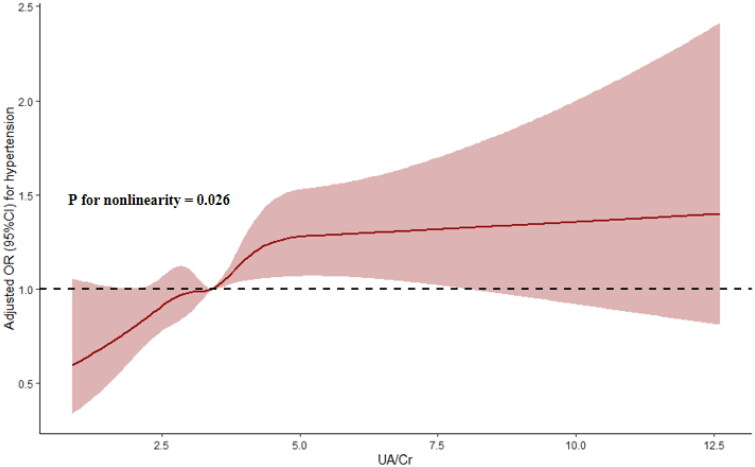
OR (95% CI) for the hypertension by the UA/Cr ratio. The association was adjusted for variables included in model 3 in Table 2. UA, uric acid; CR, creatinine; or, odd ratio; CI, confidence interval.

The RCS showed that the UA/Cr ratio was nonlinearly associated with the risk of hypertension (P for nonlinearity = 0.026) (Figure 2). Further threshold effect analysis revealed that only a UA/Cr ratio less than 5.0 was closely associated with the risk of hypertension (OR: 1.178, 95% CI: 1.086-1.278, *p* < 0.001), while a UA/Cr ratio above 5.0 was positively, but not significantly, correlated with the risk of hypertension (OR: 1.006, 95% CI: 0.918-1.103, *p* = 0.893); moreover, the 2-piecewise regression model was superior to the 1-line regression model (P for likelihood ratio test < 0.05) (Table S2).

## 4. Discussion

In this large cross-sectional study, for the first time, we found that the UA/Cr ratio was related to the prevalence of hypertension in the general population. Further subgroup and sensitivity analyses verified the robustness of these correlations in most subgroup and sensitivity analyses. Subsequent RCS and threshold effect analyses confirmed the nonlinear correlation and significant threshold effect between the UA/Cr ratio and the prevalence of hypertension, and these research results deserve further in-depth discussion.

Current evidence suggests a strong association between serum UA levels or Cr levels and renal function because reduced renal function leads to reduced urate excretion, suggesting that the negative effects of high levels of UA on some diseases may be a function of renal function [19,20]. However, two studies showed that the correlation between higher levels of UA and a greater risk of poor prognosis in patients with heart failure was significant in patients without severe CKD but not in patients with severe CKD [21,22]. In addition, due to the influence of renal function and fluctuations in blood concentrations, the use of the UA or Cr level alone for predicting renal function or other diseases is usually limited. To avoid these defects, UA and Cr levels are combined (UA/Cr ratio) to predict renal function and are more valuable than a single index, which indicates that the UA/Cr ratio may better reflect endogenous UA or Cr levels. Subsequently, the correlation between the UA/Cr ratio and other metabolic-related diseases was also explored. For example, Li et al. reported that the UA/Cr ratio was not only positively correlated with the HOMA-B score, the β cell function index and insulin resistance but also significantly independently correlated with preserved β cell function [23]. In addition, Sookoian et al. revealed a significant positive correlation between the UA/Cr ratio and NAFLD in a large cross-sectional study involving 3359 subjects [24]. Furthermore, Moriyama reported that the UA/Cr ratio was closely related to metabolic syndrome and liver function in a large cohort study from Japan [25]. Another study showed that the serum UA/Cr ratio combined with the free androgen index could predict polycystic ovary syndrome in obese women [26]. In addition to these metabolic-related diseases, several studies have confirmed that the UA/Cr ratio is closely related to cardiovascular events and mortality. For example, Casiglia et al. reported that a UA/Cr > 5.35 could independently predict cardiovascular events in men and women after an average follow-up of 126 months for 20724 participants [27]. Mazidi et al. confirmed that the UA/Cr ratio was closely related to all-cause mortality, cardiovascular mortality and cancer-related mortality in a large prospective cohort study involving 20209 people and 76.4 months of follow-up [28]. Another small sample study from China also proved that the UA/Cr ratio was closely related to all-cause mortality in elderly hemodialysis patients and that the UA/Cr ratio was more predictive than the serum uric acid concentration or the serum creatinine concentration alone [17]. In addition, Piani et al. found that a higher UA/Cr ratio during pregnancy was strongly associated with a greater risk of preeclampsia and adverse pregnancy outcomes in a cohort study of 269 women, suggesting that the UA/Cr ratio may also have clinical value in the prediction and prognosis of hypertensive disorders during pregnancy [29]. Although the above studies confirmed the association between the UA/Cr ratio and the risk of several metabolism-related diseases and mortality, its association with hypertension risk is still unknown. Only a few studies have examined the potential relationship between serum UA levels and hypertension [30–32]. However, to fill this knowledge gap, we conducted this study and found that a higher UA/Cr ratio was closely associated with a higher prevalence of hypertension, independent of renal function represented by the eGFR; moreover, the correlation between these two variables was statistically significant in most subgroup and sensitivity analyses. In addition, we found that there was a nonlinear relationship between the UA/Cr ratio and hypertension risk, and in-depth analysis revealed that a UA/Cr ratio below the threshold was positively correlated with hypertension risk, while a UA/Cr ratio above the threshold was not statistically significant. However, there was a certain positive correlation between the UA/Cr ratio and hypertension risk, which adds new evidence for the additional harm of having a high UA/Cr ratio.

Although this study achieved positive results, the underlying mechanism is still unknown. After consulting the literature, we speculated that the following mechanisms might be involved in the correlation between the UA/Cr ratio and hypertension risk. First, higher levels of UA may indicate a stronger inflammatory state and greater participation of inflammatory factors, such as C-reactive protein, interleukin-1 and tumor necrosis factor alpha, and these inflammatory cascade reactions may damage the vascular endothelium and media, thus promoting the occurrence and development of hypertension [33,34]. Second, higher levels of serum UA can lead to renal vasoconstriction enhancement, renal ischemic stimulation enhancement and immune system activation through the enhancement of oxidative stress, a decrease in the endothelial nitric oxide supply and the activation of renin angiotensin activity, which ultimately leads to an increase in the risk of hypertension [35]. Third, an increase in the serum UA concentration will cause an increase in the intracellular UA concentration, which can promote the occurrence and development of hypertension by enhancing glomerular blood pressure and systemic vascular resistance [36,37]. Fourth, high UA levels lead to excessive extracellular uric acid deposition in plaques and the formation of crystals in peripheral blood vessels, resulting in the destruction of the intima-media structure and the occurrence of hypertension [38]. Fifth, as mentioned above, higher UA/Cr ratios are related to worse β-cell function, which indicates that UA and Cr may participate in insulin resistance and metabolic syndrome through this mechanism and indirectly increase the prevalence of hypertension [23]. Sixth, hyperuricemia and gout caused by high levels of UA may lead to excessive oxidative stress and increased apoptosis, which further damages endothelial function and the structure of vascular media and ultimately leads to vasomotor dysfunction and arterial stiffness [36,39]. In addition to the above possible mechanisms, additional potential mechanisms need to be further revealed by future studies.

Because this was a cross-sectional study based on the survey population, our study inevitably had several limitations. For example, we did not analyze the correlation between the UA/Cr ratio and the incidence of hypertension during follow-up. In addition, due to the lack of genetic data, we could not determine the causal association between the UA/Cr ratio and hypertension. Furthermore, since the hypertensive individuals in this study were from a community-based population in China and because the hypertension data were not obtained from the hospital electronic medical records system, which meant that we were unable to determine the maximum blood pressure level or the etiology of hypertension in these patients, we were unable to grade hypertension or categorize the hypertension etiology. Additionally, as this was an epidemiological investigation, the study design was consistent with that of most epidemiological studies, which meant that we did not perform propensity score-matched analyses, but we adjusted for all confounders in our regression analyses; therefore, this approach could reduce bias in the results. Finally, we could not avoid missing some parameters that might affect the results, such as genetic susceptibility and environmental factors.

## 5. Conclusion

In this large-scale cross-sectional study based on a population survey, we revealed a strong association between the UA/Cr ratio and the prevalence of hypertension, suggesting that we should incorporate the UA/Cr ratio into the routine assessment of hypertension risk.

## Supplementary Material

Supplemental Material

## Data Availability

All raw data and materials included in this study are publicly available on the CHNS website.
