# Supplementary material for: A nonlinear correlation between the serum uric acid to creatinine ratio and the prevalence of hypertension: a large cross-sectional population-based study
Source: Ren Fail. 2024 Jan 8;46(1):2296002. doi: 10.1080/0886022X.2023.2296002 (PMC10776046; doi:10.1080/0886022X.2023.2296002)
Supplement: Supplemental Material [file IRNF_A_2296002_SM1633.pdf]

SUPPLEMENT

Table S1. Baseline characteristics after excluding participants with eGFR < 30 ml/min/1.73 m^2.

Table S2. Threshold effect analysis of the UA/Cr ratio on hypertension.

Information Classification: General

Table S1. Baseline characteristics after excluding participants with eGFR < 30 ml/min/1.73 m^2.

	Non-hypertension	Hypertension	P value
Age, years	46.94 ± 14.40	59.13 ± 12.81	< 0.001
Sex, male, n (%)	2813 (46.00%)	1210 (49.80%)	0.001
Smoking status, n (%)			< 0.001
Now	1701 (27.80%)	674 (27.70%)	
Ever	155 (2.50%)	126 (5.20%)	
Never	4262 (69.60%)	1629 (67.00%)	
Diabetes, n (%)	456 (7.50%)	466 (19.20%)	< 0.001
Hypoglycemic drugs, n (%)	75 (1.20%)	140 (5.80%)	< 0.001
Body mass index, kg/m^2	22.87 ± 3.23	24.68 ± 3.70	< 0.001
Systolic blood pressure, mmHg	116.09 ± 11.19	144.78 ± 18.03	< 0.001
Diastolic blood pressure, mmHg	75.60 ± 7.51	90.76 ± 10.88	< 0.001
Triglyceride, mmol/L	1.17 (0.80, 1.80)	1.51 (1.01, 2.33)	< 0.001
Total cholesterol, mmol/L	4.77 ± 0.98	5.11 ± 1.02	< 0.001
LDL-C, mmol/L	2.91 ± 0.95	3.16 ± 1.02	< 0.001
HDL-C, mmol/L	1.45 ± 0.44	1.41 ± 0.60	0.004
Apolipoprotein A1, g/L	1.15 ± 0.35	1.17 ± 0.45	0.051
Apolipoprotein B, g/L	0.88 ± 0.26	0.99 ± 0.28	< 0.001
CR, µmol/L	85.57 ± 14.78	90.49 ± 17.95	< 0.001
UA, µmol/L	298.90 ± 103.90	332.56 ± 105.48	< 0.001
Fasting plasma glucose, mmol/L	5.26 ± 1.29	5.78 ± 1.79	< 0.001
Hemoglobin A1c, %	5.53 ± 0.79	5.89 ± 1.14	< 0.001
Hs-CRP, mg/L	1.00 (0, 2.00)	2.00 (1.00, 3.00)	< 0.001
eGFR, ml/min/1.73m^2	84.16 ± 17.92	76.21 ± 16.32	< 0.001
UA/Cr	3.51 ± 1.50	3.71 ± 1.13	< 0.001

Data were expressed as mean ± SD, median (interquartile range), or n (%). eGFR, estimated glomerular filtration rate; LDL-C, low-density lipoprotein cholesterol; HDL-C, high-density lipoprotein cholesterol; CR, creatinine; UA, uric acid; Hs-CRP, high-sensitivity C-reactive protein.

Information Classification: General

Table S2. Threshold effect analysis of the UA/Cr ratio on hypertension.

		OR (95% CI)	P value
1-line regression model		1.053 (1.013, 1.094)	0.008
Inflection point (K)		5.0	
2-piecewise regression model	< K segment	1.178 (1.086, 1.278)	< 0.001
	> K segment	1.006 (0.918, 1.103)	0.893
P for log likelihood ratio test		< 0.05	

The analysis was adjusted for variables included in model 3 in Tables 2. UA, uric acid; CR, creatinine; OR, odd ratio; CI, confidence interval.

Information Classification: General
